# Synbiotic as an ameliorating factor in the health-related quality of life in women with polycystic ovary syndrome. A randomized, triple-blind, placebo-controlled trial

**DOI:** 10.1186/s12905-023-02868-1

**Published:** 2024-01-03

**Authors:** Zahra Hariri, Zahra Yari, Sedighe Hoseini, Khadijeh Abhari, Golbon Sohrab

**Affiliations:** 1 Student Research Committee, Department of Clinical Nutrition and dietetics, Faculty of Nutrition Sciences and Food Technology, Shahid Beheshti University of Medical Sciences, Tehran, Iran; 2 Department of Nutrition Research, National Nutrition and Food Technology Research Institute, Faculty of Nutrition Sciences and Food Technology, Shahid Beheshti University of Medical Sciences, Tehran, Iran; 3 Department of Obstetrics and Gynecology, Preventative Gynecology Research Center, Shahid Beheshti University of Medical Sciences, Tehran, Iran; 4 Department of Food Sciences and Technology, Faculty of Nutrition Sciences and Food Technology, National Nutrition and Food Technology Research Institute, Shahid Beheshti University of Medical Sciences, Tehran, Iran; 5 Clinical Nutrition and dietetics Department, Faculty of Nutrition Sciences and Food Technology, National Nutrition and Food Technology Research Institute, Shahid Beheshti University of Medical Sciences, Tehran, Iran

**Keywords:** Polycystic ovary syndrome, Health-related quality of life, Synbiotic, Bacillus coagulans

## Abstract

**Background:**

There are complicated mechanisms that link the disruption of the gut microbiome to the symptoms and complications of polycystic ovary syndrome (PCOS). In this study, an attempt was made to assess the effects of synbiotics on the health-related quality of life (HRQoL) in women with PCOS .

**Methods:**

Fifty-six women with PCOS were enrolled in a triple-blind controlled trial for 12 weeks. They were randomly assigned to receive a daily 2-gram synbiotic sachets (containing Bacillus coagulans (GBI-30), Lactobacillus rhamnosus, Lactobacillus helveticus, and fructooligosaccharide) (n = 28) or placebo (n = 28). To evaluate the impact on the HRQoL, participants were required to fill 26-Item Polycystic Ovary Syndrome Health-Related Quality of Life Questionnaire (PCOSQ-26), 12-Item Short-Form Health Survey (SF-12) and Perceived Stress Scale (PSS-10) pre and post the intervention.

**Results:**

Finally, statistical analyses were performed on 52 participants who finished the trial. Synbiotic supplementation improved the scores of emotional (*P* = 0.044), body hair (*P* = 0.016), weight (*P* = 0.033) and infertility domains (*P* = 0.027) of PCOSQ-26 compared to placebo group. The physical score within SF-12 also had a significant enhancement (*P* = 0.035). No significant improvement was seen in the PSS-10 score at the end of the trial.

**Conclusion:**

This study illustrated the advantageous effects of synbiotics on the health-related quality of life in women with PCOS. Further studies are required to confirm our findings.

**Trial registration:**

http://www.irct.ir: IRCT20211108053007N1; date of registration: 14/02/2023.

## Background

Polycystic ovary syndrome (PCOS), as a chronic endocrine disorder, can affect many aspects of young women’s lives [1]. According to the Rotterdam criteria, which is one of the key diagnostic indicators of polycystic ovary syndrome, about 2 out of every 10 women of reproductive age in Iran suffer from PCOS [2, 3]. These women experience a wide range of symptoms, including amenorrhea, oligomenorrhea, polymenorrhea, delayed ovulation, anovulation, infertility weight gain, obesity, acne, alopecia, and hirsutism [4, 5]. Moreover, PCOS is a risk factor for endometrial cancer [6]. Apart from physical complications, women with polycystic ovary syndrome are more likely to suffer from mental and behavioral disorders [7]. Considerable rates of depression, anxiety, panic attacks, eating disorder, bipolar disorder, mood swings and psychosis have been reported among these people [8, 9]. Although the exact pathophysiology of PCOS-related mental disorders is not yet known, it seems that hirsutism, acne, overweight and obesity, and finally infertility have a direct relationship with these complications [9, 10]. As a consequence of unfavorable physical and mental conditions, women with PCOS may have a poor health-related quality of life (HRQoL) [11, 12].

Several lines of evidence suggest a relationship between the gut microbiome and PCOS [13–15]. A defective microbiome can influence the progression of PCOS through hyperandrogenism, gut-brain axis disorder, impaired epithelial receptor–mediated signaling and increased secretion of inflammatory cytokines [16–19]. Administration of prebiotics and probiotics can be effective solutions for treating dysbiosis. Synbiotics, as a combination of prebiotics and probiotics, have an intensified impact on selectively stimulating the growth and activation of beneficial gut bacteria than either alone [20]. Once probiotics consumed, they go through the challenging digestive tract, so if they survive stomach and bile acid, digestive enzymes and antibacterial molecules, they can colonize in the large intestine and improve the microbiome [21]. This is while, the addition of prebiotics as indigestible and growth-promoting carbohydrates can improve the survival and emplacement of probiotics [22].

A recent meta-analysis conducted in PCOS women indicated beneficial effects of probiotics, prebiotics and synbiotics on glycemic status, insulin resistance and lipid profile [23]. Although so far many interventions have measured the effect of probiotics/synbiotics on the HRQoL in various disorders, none of them have measured this effect among women with PCOS. The Polycystic Ovary Syndrome Health-Related Quality of Life Questionnaire (PCOSQ) was developed in 1998 to accurately evaluate the unique symptoms of PCOS [24]. In addition, the 12-Item Short-Form Health Survey (SF-12) and Perceived Stress Scale (PSS-10) are widely used to evaluate overall health (physical and mental) and stress levels in facing life situations, respectively [25, 26]. In a study that examined the PCOSQ-26 and SF-36 scores among 3 groups of infertile PCOS, non-PCOS infertile and fertile PCOS participants, the lowest score was observed among infertile PCOS women followed by fertile PCOS. They concluded that apart from the impact of infertility, PCOS alone plays an efficacious role in reducing HRQoL [27].

Considering the reported beneficial effects of probiotic/synbiotic on PCOS, we hypothesized that synbiotic supplementation could improve HRQoL in these women, and therefore, for the first time, the present study aimed to test this hypothesis.

## Materials and methods

### Study design

In this triple-blind, randomized clinical trial, women with polycystic ovary syndrome were randomly divided into synbiotic or placebo groups for 12 weeks. During this period, the synbiotic group received daily 2-gram sachets containing ${10}^{11}$ spores of Bacillus coagulans (GBI-30), ${10}^{10}$ colony-forming units (CFU) of Lactobacillus rhamnosus, ${10}^{10}$ CFU of Lactobacillus helveticus, 500 mg of fructooligosaccharides and 0.7% natural orange flavoring and the placebo group received daily 2-gram sachets filled with starch and 0.7% natural orange flavoring similar in packaging, texture and taste and were labeled with two letters A and B to blind the researchers, statistician and participants. Both types of supplement were manufactured and labelled by Pardis Roshd Mehregan company (Shiraz, Iran). Eligible participants were block stratified randomized based on BMI (< 25 kg/m2 or ≥ 25 kg/m2), use of menstrual-regulating hormonal drugs (yes or no) and metformin (yes or no). All the steps of this study were carried out according to the Declaration of Helsinki and Good Clinical Practice guidelines. Monthly phone call or virtual communication was made to follow up the regular intake of supplements, and if participants reported any side effects or received less than 90% of their supplement, they were excluded from the study.

### Participants

This intervention was carried out between February and May 2023. The participants were invited by a gynecologist (S.H.) from women referring to the obstetrics and gynecology clinic of Ayatollah Taleghani Hospital affiliated to Shahid Beheshti University of Medical Sciences in Tehran. Participants assignment to interventions was performed by the main researcher (ZH). The inclusion criteria were: newly diagnosed PCOS based on the Rotterdam criteria [28], age 18–45, body mass index (BMI) between 18.5 and 35. Exclusion criteria were: following a weight loss diet or any special diet, smoking, receiving any antibiotics or products containing probiotics/synbiotics in the past month, diagnosis of pregnancy, heart or kidney failure, kidney disease, liver disease, hypothyroidism or hyperthyroidism, malignancy, hyperprolactinemia and any infectious or inflammatory disease.

### Anthropometric assessment

General characteristics and anthropometric measurements of participants were determined at the beginning and end of the study. Weight and height were measured using a mechanical column scale, and waist and hip circumference were measured via a tape measure. BMI (kg/m^2^) was calculated through dividing the weight (kg) by the square of the height (m^2^). In this study, participants have been advised not to change their diet or exercise in this study.

### Questionnaires

Participants were asked to fill out three questionnaires, namely PCOSQ, SF12 and PSS10 at the beginning and end of the study. The main researcher (ZH) supervised full completion of the questionnaires. These questionnaires are described and interpreted as follows:

#### Polycystic ovary syndrome health-related quality of life questionnaire (PCOSQ-26)

The validated Iranian version of the 26-item PCOSQ prepared by Amini et al. [29] was applied. This questionnaire contained emotional, body hair, weight, infertility, and menstrual problems domains, and each question was graded on a 7-item Likert scale so that a score of 7 indicated the best and a score of 1 indicated the weakest performance. At last, to make a better comparison, the score of each domain was calculated out of 100.

#### 12-item short-form health survey (SF-12)

This 12-item questionnaire is a short and modified version of 36-item form, and its validity and reliability have been evaluated by Montazeri et al. in Iran [30]. This questionnaire is divided into two subscales, physical and mental. The physical subscale includes physical functioning, role limitation due to physical problems, perception of general health, physical pain, and the mental subscale includes role limitation due to mental-psychological problems, vitality, mental state and social functioning. To score this questionnaire, the number in front of each option indicates the score of that option. Questions No. 1, 8, 10, and 11 are scored inversely. Afterward, mental component scale (MCS) and physical component scale (PCS) scores are obtained as final results.

#### Perceived stress scale (PSS-10)

The perceived stress scale was first presented by Cohen et al. and according to their description: “The PSS measures the degree to which situations in one’s life are appraised as stressful” [31]. Since 10-item-PSS has more desirable psychometric characteristics than other versions [32], we used PSS-10 in an Iranian version that was validated by Marofizadeh et al. [33]. The scoring of each question was from 0 (lowest) to 4 (highest) as the Likert method. Questions NO. 4, 5, 7 and 8 were scored in reverse.

### Sample size

The determination of the sample size was based upon the standard deviation attributable to emotional domain in PCOSQ-26 [34]. By assuming a score difference of 9 (d), type one error (α) of 0.05 and type two error (β) of 0.20 (power = 80%), and placing them in the formula (n = 2S^2^(Z_1−α/2_+Z_1−β_)^2^/d^2^), we obtained the sample size of 28 in each group (including 10% dropout).

### Statistical analysis

Primitively, the Kolmogorov–Simonov test was performed to ensure the normal distribution of the variables. Intra-group and inter-group comparisons were done using paired t-tests and independent t-tests, respectively. The analysis of covariance (ANCOVA) test was used to investigate the interaction effect of possible confounders. Statistical Package for Social Science software version 26 (SPSS Inc., Chicago, Illinois, USA) was utilized to operate the statistical analyses. Quantitative variables were reported as mean and standard deviation (SD) and qualitative variables were reported as frequency (percentage). *P* < 0.05 represented statistical significance. As a means to evaluate the internal consistency for each questionnaire, Cronbach’s alpha was calculated for the initial and final outcomes.

## Results

**Fig. 1:**
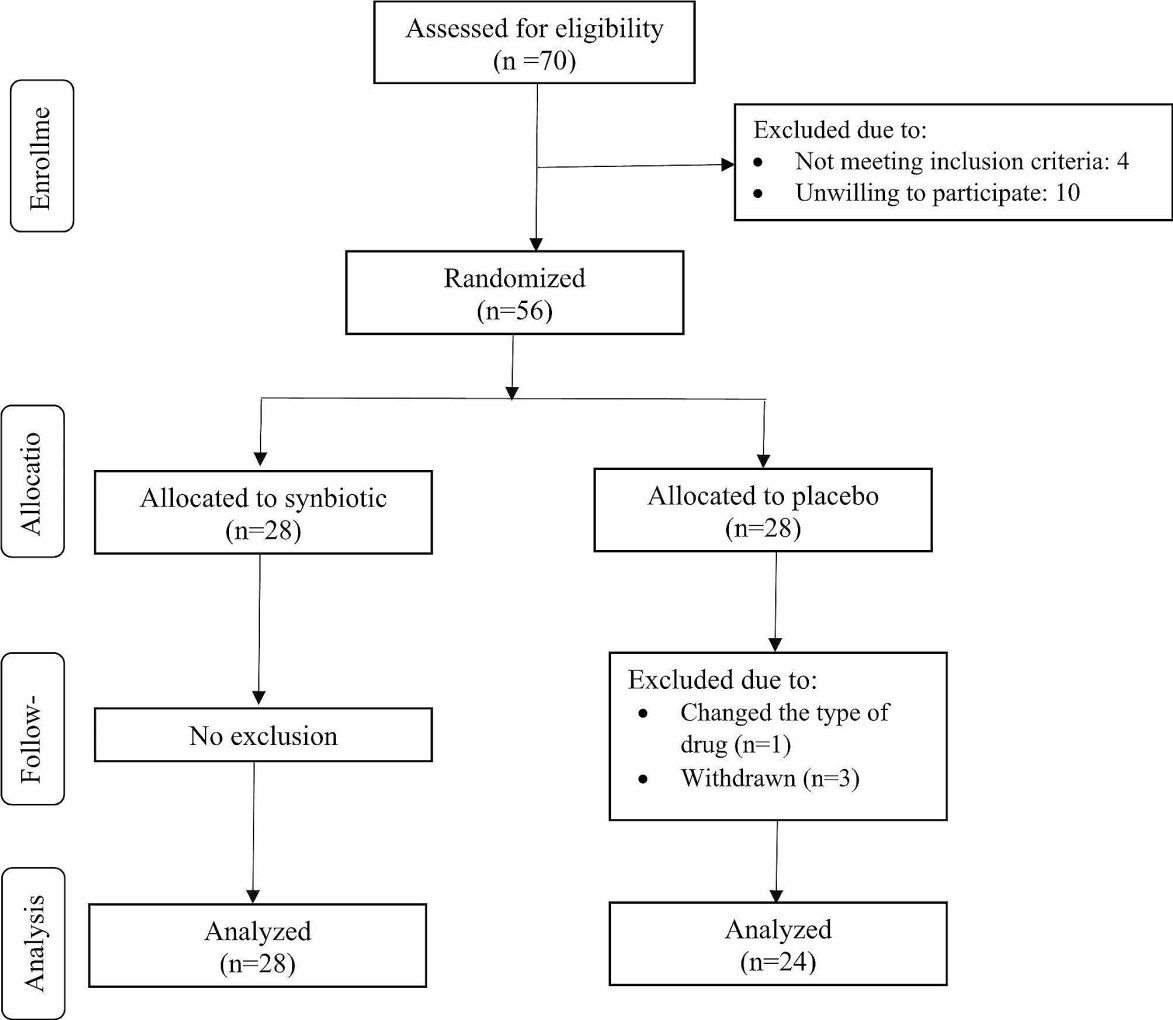
Flow diagram of participant’s recruitment

From February to May 2023, 70 people were evaluated to participate in the study, and 56 of them met the eligibility criteria. In the following, 4 people left the placebo group due to unwillingness to continue and changes in the taking drugs. Finally, the remaining 52 individuals were subjected to statistical analysis (Fig. 1).

With a view to Table 1, there was no significant difference in the initial characteristics of the participants of the two groups. Similarly, no meaningful difference in anthropometric indices (weight, BMI, WC and HC) between the groups was observed prior to or after intervention as described in Table 2.

**Table 1:** Baseline characteristics of participants

Characteristics	Intervention group N = 28	Placebo group N = 24	*p*-Value
Age (years)	28.42 ± 6.10	32.75 ± 15.99	0.222
Height (cm)	163.08 ± 4.51	163.06 ± 4.24	0.983
Marital status (%)			
Single Married Widow Divorced	15 (53.6%) 12 (42.9%) 0 (0%) 1 (3.6%)	12 (50.0%) 9 (37.5%) 0 (0%) 3 (12.5)	0.481
History of previous pregnancy (%)			
Yes No	3 (10.7%) 25 (89.3%)	3 (12.5%) 21 (87.5%)	0.841
Duration of PCOS (year)	9.80 ± 6.21	7.48 ± 4.87	0.152
On antidepressant drugs (%)			
Yes No	4 (14.3%) 24 (85.7%)	3 (12.5%) 21 (87.5%)	0.851
On antianxiety drugs (%)			
Yes No	2 (7.1%) 26 (92.9%)	3 (12.5%) 20 (83.3%)	0.431
On menstrual-regulating drugs (%)			
Yes No	5 (55.6%) 23 (53.5%)	4 (44.4%) 20 (46.5%)	1.000
On metformin (%)			
Yes No	3 (42.9%) 25 (55.6%)	4 (57.1%) 20 (44.4%)	0.531

BMI: Body mass index; PCOS: polycystic ovarian syndrome.

Quantitative variables are mean ± SD and qualitative variables are frequency (percentage).

**Table 2:** Anthropometric indices at the beginning and after 12 weeks of intervention in women with polycystic ovary syndrome

Characteristics	Intervention group N = 28	Placebo group N = 24	P**
Weight (kg)			
Baseline End of trial Change P*	67.17 ± 11.84 66.92 ± 12.55 -0.25 ± 3.24 0.683	66.10 ± 11.45 65.18 ± 10.42 -0.06 ± 2.42 0.903	0.742 0.603 0.820
BMI (kg/m^2^)			
Baseline End of trial Change P*	25.19 ± 4.09 24.09 ± 4.22 -0.10 ± 1.26 0.676	24.86 ± 4.33 24.48 ± 3.73 -0.05 ± 0.90 0.779	0.780 0.592 0.882
WC (cm)			
Baseline End of trial Change P*	83.25 ± 10.92 81.96 ± 11.23 -0.79 ± 5.63 0.492	82.66 ± 10.24 82.17 ± 10.62 0.00 ± 5.86 1.000	0.843 0.946 0.642
HC (cm)			
Baseline End of trial Change P*	103.46 ± 9.08 102.74 ± 10.74 -0.51 ± 6.02 0.664	102.75 ± 8.68 101.30 ± 8.17 -1.04 ± 3.48 0.165	0.776 0.602 0.716

P*: *p*-values for comparison within groups via paired t-test.

P**: *p*-values for comparison between groups via independent t-test.

Abbreviations: CI: confidence interval; BMI: body mass index; WC: waist circumference; HC: hip circumference.

Table 3 signifies the PCOSQ outcomes. Emotional (*P* = 0.029) and infertility (*P* = 0.023) scores significantly increased within the synbiotic group. In addition, based on the analysis of covariance, emotional (*P* = 0.044), body hair (*P* = 0.016), weight (*P* = 0.033) and infertility (*P* = 0.027) scores significantly increased after synbiotic supplementation in comparison with the placebo group. No significant improvement was seen in the menstrual problems after 12 weeks of trial. Cronbach’s alpha for PCOSQ-26 in the synbiotic (pre-intervention: 0.857 and post-intervention: 0.906) and placebo groups (pre-intervention: 0.926 and post-intervention: 0.946) indicated good internal consistency.

**Table 3:** The domains of PCOSQ-26 questionnaire at the beginning and after 12 weeks of intervention in people with polycystic ovary syndrome

Variables	Intervention group N = 28	Control group N = 24	95% CI		P**	P***
			Lower	Upper		
Emotions domain						
Baseline End of trial Change P*	55.67 ± 11.00 61.60 ± 13.71 5.93 ± 13.60 0.029	57.36 ± 19.72 65.84 ± 20.93 8.48 ± 13.41 0.050	-10.89 -13.97 -10.10	7.51 5.48 5.00	0.711 0.386 0.501	0.044
Body hair domain						
Baseline End of trial Change P*	58.18 ± 19.87 55.51 ± 19.78 1.32 ± 17.41 0.690	62.14 ± 27.83 70.95 ± 23.82 8.80 ± 16.63 0.016	-21.29 -27.58 -17.01	5.37 -3.29 2.04	0.236 0.014 0.121	0.016
Weight domain						
Baseline End of trial Change P*	54.18 ± 19.87 55.51 ± 19.78 1.32 ± 17.41 0.690	62.14 ± 27.83 70.95 ± 23.82 8.80 ± 16.63 0.016	-21.29 -27.58 -17.01	5.37 -3.29 2.04	0.236 0.014 0.121	0.033
Infertility domain						
Baseline End of trial Change P*	76.02 ± 20.01 82.52 ± 17.31 6.50 ± 14.22 0.023	76.48 ± 16.41 75.14 ± 20.35 -1.33 ± 15.21 0.670	-10.77 -3.11 -0.36	9.83 17.86 16.05	0.928 0.164 0.061	0.027
Menstrual problems						
Baseline End of trial Change P*	54.20 ± 16.69 58.03 ± 16.25 3.82 ± 14.15 0.164	60.56 ± 22.15 65.62 ± 22.26 5.05 ± 24.78 0.328	-17.19 -18.34 -12.27	4.48 3.16 9.81	0.244 0.163 0.823	0.389
Cronbach’s alpha						
Baseline End of trial	0.857 0.906	0.926 0.946				

P*: *p*-values for comparison within groups via paired t-test.

P**: *p*-values for comparison between groups via independent t-test.

P***: *p*-values based on an ANCOVA model adjusted with baseline value of the outcome, age, marital status, PCOS duration and mean changes in BMI, waist circumference and hip circumference.

CI: confidence interval

SF-12-related results are presented in Table 4. Physical score in the synbiotic group, increased significantly compared to the placebo group (*P* = 0.035). However, this increase was insignificant compared to the beginning of the study. Also, from a statistical point of view, there was no improvement in the mental domain score either in the intra-group or inter-group analysis. Good internal consistency was observed in synbiotic (pre-intervention: 0.806 and post-intervention: 0.882) and placebo groups (pre-intervention: 0.903 and post-intervention: 0.940) based on Cronbach’s alpha.

**Table 4:** The domains SF-12 questionnaire at the beginning and after 12 weeks of intervention in people with polycystic ovary syndrome

Variables	Intervention group N = 28	Control group N = 24	95% CI		P**	P***
			Lower	Upper		
Physical score						
Baseline End of trial Change	66.75 ± 12.61 68.95 ± 14.23 2.19 ± 10.79	65.54 ± 17.94 73.07 ± 18.91 7.53 ± 18.02	-7.33 -13.36 -13.47	9.76 5.12 2.80	0.777 0.375 0.194	
P*	0.291	0.052				
Mental score						
Baseline End of trial Change	55.06 ± 13.31 55.00 ± 15.72 -0.37 ± 13.96	54.72 ± 18.17 59.16 ± 19.29 4.44 ± 13.92	-8.55 -13.92 -12.67	9.23 5.58 3.04	0.939 0.395 0.224	
P*	0.891	0.132				
Cronbach’s alpha						
Baseline End of trial	0.806 0.882	0.903 0.940				

P*: *p*-values for comparison within groups via paired t-test.

P**: *p*-values for comparison between groups via independent t-test.

P***: *p*-values based on an ANCOVA model adjusted with baseline value of the outcome, age, marital status, PCOS duration and mean changes in BMI, waist circumference and hip circumference.

CI: confidence interval

Table 5 describes the PSS-10 by groups before and after the intervention. There was no statistically significant improvement in the stress score within and between groups. Cronbach’s alpha represented good internal consistency in synbiotic (pre-intervention: 0.828 and post-intervention: 0.847) and placebo groups (pre-intervention: 0.889 and post-intervention: 0.894).

**Table 5:** Perceived stress scale measurement items (PSS-10) at baseline and after the 12-week intervention in subjects with polycystic ovary syndrome

PSS-10 Descriptive statistics	assessment time	Intervention group N = 28	Control group N = 24	P**	P***
In the Last Month, How Often Have You:					
1- Been upset because of something that happened unexpectedly?	Baseline End of trial Change P*	2.28 ± 1.01 2.64 ± 1.12 0.35 ± 1.19 0.125	2.66 ± 1.27 2.50 ± 0.97 -0.16 ± 1.09 0.461	0.236 0.631 0.107	0.161
2- Felt that you were unable to control the important things in your life?	Baseline End of trial Change P*	2.50 ± 0.95 2.28 ± 1.04 -0.21 ± 0.99 0.264	2.54 ± 1.28 2.45 ± 1.25 -0.08 ± 0.113 0.723	0.894 0.591 0.660	0.752
3- Felt nervous and “stressed”?	Baseline End of trial Change P*	2.46 ± 1.07 2.35 ± 1.06 -0.10 ± 1.16 0.631	2.70 ± 1.04 2.70 ± 1.12 0.00 ± 1.21 1.00	0.411 0.252 0.747	0.669
4- Felt confident about your ability to handle your personal problems? ^R^	Baseline End of trial Change P*	1.82 ± 1.02 1.75 ± 0.92 -0.07 ± 1.15 0.745	1.50 ± 1.06 1.50 ± 1.02 0.00 ± 1.17 1.00	0.272 0.360 0.826	0.735
5- Felt that things were going your way? ^R^	Baseline End of trial Change P*	2.35 ± 0.86 2.17 ± 0.81 -0.17 ± 1.05 0.379	2.12 ± 1.03 1.87 ± 1.07 -0.25 ± 1.42 0.398	0.384 0.254 0.837	0.342
6- Found that you could not cope with all the things that you had to do?	Baseline End of trial Change P*	2.11 ± 0.97 1.96 ± 0.79 -0.11 ± 0.89 0.523	2.00 ± 1.06 2.29 ± 1.23 0.29 ± 1.12 0.216	0.699 0.254 0.160	0.051
7- Been able to control irritations in your life? ^R^	Baseline End of trial Change P*	2.03 ± 0.79 1.89 ± 0.91 -0.14 ± 0.97 0.443	1.70 ± 0.95 1.83 ± 0.91 0.12 ± 1.07 0.575	0.183 0.816 0.350	0.956
8- Felt that you were on top of things? ^R^	Baseline End of trial Change P*	2.32 ± 0.77 2.14 ± 0.89 -0.17 ± 0.90 0.306	2.12 ± 0.94 1.95 ± 0.99 -0.16 ± 1.09 0.461	0.414 0.485 0.966	0.630
9- Been angered because of things that were outside of your control?	Baseline End of trial Change P*	2.25 ± 0.92 2.35 ± 0.95 0.10 ± 0.99 0.573	2.62 ± 0.92 2.37 ± 1.24 -0.25 ± 0.94 0.207	0.152 0.954 0.192	0.482
10- Felt difficulties were piling up so high that you could not overcome them?	Baseline End of trial Change P*	2.39 ± 1.10 2.46 ± 1.07 0.07 ± 1.05 0.722	2.41 ± 1.34 2.12 ± 1.32 -0.29 ± 1.23 0.258	0.944 0.313 0.257	0.118
Total score	Baseline End of trial Change P*	22.55 ± 5.97 22.03 ± 6.25 -0.37 ± 5.74 0.740	22.41 ± 7.81 21.62 ± 8.12 -0.79 ± 5.94 0.520	0.943 0.838 0.798	0.811
Cronbach’s alpha Baseline End of trial	0.828 0.847	0.889 0.894			

^R^ reverse scored item

P*: *p*-values for comparison within groups via paired t-test.

P*: *p*-values for comparison between groups via independent t-test.

P***: *p*-values based on an ANCOVA model adjusted with baseline value of the outcome, age, marital status, PCOS duration and mean changes in BMI, waist circumference and hip circumference.

## Discussion

In this 12-week placebo-controlled intervention, the effects of synbiotic supplementation containing Bacillus coagulans (GBI-30), Lactobacillus rhamnosus, Lactobacillus acidophilus, and fructooligosaccharide have been assessed on the HRQoL among women with PCOS. As a resultant, emotional, body hair, weight and infertility domains of PCOSQ-26 and the physical domain of SF-12 improved, but no significant changes in the PSS-10 items and mental domain of SF-12 were seen. In fact, the result obtained in this study was in line with our hypothesis about the positive effects of synbiotic supplementation on the improvement of the emotional domain of the PCOSQ-26, which was considered as the primary outcome of the study.

Several investigations have reported a decreased quality of life in women with PCOS [27, 35–37]. Coffey et al., [36] by comparing the HRQoL of PCOS participants with healthy people, found worse scores in SF-36 and all PCOSQ-26 domains among PCOS participants. Thereafter, the comparison was made between women with PCOS and individuals with severe health conditions, including asthma, epilepsy, diabetes, back pain, arthritis and coronary heart disease, only through the SF-12, and as a result, PCOS participants showed worse scores in the mental domain and the same or better scores in physical domain [36].

Since this clinical trial was the first of its kind, there hasn’t been quite an identical example so far. Type 2 diabetes mellitus and PCOS share similarities in their main underlying pathophysiology, which is insulin resistance. In agreement with our findings, during a 3-month intervention with synbiotic capsules in type 2 diabetes mellitus patients, a significant improvement was achieved in the scores of the diabetes specific HRQoL questionnaire [38]. Likewise, in another placebo-controlled study that was conducted on pre-diabetic people, supplementation with Lactobacillus rhamnosus HN001 was able to meaningfully improve the social functioning and mental state domains of SF-12 [39]. Unlike the two mentioned studies, in a 6-month study based on both probiotic and prebiotic administration in pre-diabetics, no significant difference found in SF-36 and depression, anxiety and stress scale (DASS-21) scores. However, there was no betterment in any of the metabolic or anthropometric outcomes, either [40]. Positive impact of probiotic/synbiotic on the HRQoL was evident in healthy people [41] and in patients with IBS [42], systemic sclerosis [43], cirrhosis [44] and minor digestive symptoms [45].

The quality of life can be considered as a total of physical and mental subscales, and the improvement of each leads to overall HRQoL improvement. Shoaei et al., achieved a significant reduction in fasting blood glucose (FBG) and insulin during a 8-week placebo-controlled supplementation with probiotics in PCOS cases [46]. Apart from the glycemic status, Karamali et al. proved the ameliorating effect of probiotics on total testosterone, sex hormone-binding globulin (SHBG), modified Ferriman Gallwey (hirsutism indicator tool), high-sensitivity C-reactive protein (hs-CRP), total antioxidant capacity (TAC) and malondialdehyde (MDA) levels in women with PCOS [47]. Similar anti-inflammatory effect was also remarkable in our previous synbiotic-based study among PCOS participants [48].

Altered gut microbiota in women with PCOS has been reported in several studies, which can cause a decrease in short-chain fatty acids (SCFA) production [15, 49, 50]. Lack of SCFAs can endanger the integrity of the gut barrier and triggers systemic inflammation and insulin resistance through inducing endotoxemia [51]. Moreover, due to the regulatory role of SCFAs for gut-brain axis mediators, and thus the regulation of androgen secretion, its depletion may lead to androgens disarray [52]. Excluding the SCFA functions, there appears to be a direct interaction between gut microbiota and sex hormones. As per human studies, Collinsella, Prevotella, Bacteroides and Streptococcus showed a positive relationship and Bifidobacterium and Fusicatenibacter showed a negative relationship with circulating testosterone levels. The mentioned pathways can explain the probable linkages between dysbiosis and the physical symptoms of PCOS, which are rooted in insulin resistance [53], hyperandrogenism [54] and inflammation [55]. Accordingly, supplementation with probiotics as replaceable beneficial bacteria, prebiotics as bacteria consumables, and in combination, synbiotics, can help improve the overall condition of these patients.

Although there are numerous reports on the interrelationship between mental health and gut microbiome [56], this matter hasn’t been investigated in women with PCOS. Dysbiosis can affect the hypothalamus-pituitary-adrenal (HPA) system through inflammatory factors [57]. Thereby, this system can arouse stress response by releasing the cortisol hormone [58]. The stress resulting from this pathway can deeply lead to depression and anxiety disorders [59].

Ostadmohammadi et al. showed that the co-administration of vitamin D every 2 weeks plus probiotics for 12 weeks could lead to an increase in the Beck depression inventory (BDI), general health questionnaire (GHQ) and DASS scores; This intervention was also able to significantly reduce total testosterone, hs-CRP and MDA levels and an increase total antioxidant capacity and total glutathione (GSH) levels [60]. The examined questionnaires and the findings of this study were exactly in line with Jamilian’s intervention, in which the same dose of probiotic supplement was given along with selenium supplement for 12 weeks [61].

The main strength of this study is being the first to examine the synbiotics effects on the HRQoL of women with polycystic ovaries. Besides, in order to avoid possible biases, this study was designed as a randomized, triple-blind, placebo-controlled trial. This study had also some limitations. First, the type and severity of polycystic ovary syndrome was not taken into consideration in collecting the samples. Second, physical activity was not measured as a confounding factor. And finally, participants’ life changes were not the same, and during the intervention, events may have happened to each participant that affected their answers.

## Conclusion

In summation, 12-week supplementation with synbiotics could noticeably improve the emotional, body hair, weight, infertility and general physical health status of women with polycystic ovary syndrome.

## Data Availability

The analyzed data from this clinical intervention is included in the article. However, the data generated are not publicly available due to agreements made with the sponsoring committee, but are available from the corresponding author upon reasonable request.

## Abbreviations

PCOS: Polycystic Ovary Syndrome
HRQoL: Health-Related Quality of Life
PCOSQ-26: 26 Item Polycystic Ovary Syndrome Health-Related Quality of Life Questionnaire
SF-12: 12-Item Short-Form Health Survey
PSS-10: Perceived Stress Scale
CFU: Colony-Forming Units
BMI: Body Mass Index
MCS: Mental Component Scale
PCS: Physical Component Scale (PCS)
ANCOVA: Analysis of Covariance
SPSS: Statistical Package for Social Science
SD: Standard deviation
DASS-21: 21-Item Depression, Anxiety and Stress Scale
SHBG: Sex Hormone-Binding Globulin
hs-CRP: High-Sensitivity C-Reactive Protein, TAC:Total Antioxidant Capacity
MDA: Malondialdehyde
DHEA-S: Dehydroepiandrosterone-Sulfate
TC: Total Cholesterol
TG: Triglyceride
LDL-C: Low Density Lipoprotein Cholesterol
HDL-C: High Density Lipoprotein Cholesterol (HDL-C)
SCFA: Short-Chain Fatty Acids
HPA: Hypothalamus-Pituitary-Adrenal
IU: International Unit
BDI: Beck Depression Inventory
GHQ: General Health Questionnaire
GSH: Total Glutathione
